# Tuning gold-based surface functionalization for streptavidin detection: A combined simulative and experimental study

**DOI:** 10.3389/fmolb.2022.1006525

**Published:** 2022-11-28

**Authors:** Sutapa Dutta, Mariacristina Gagliardi, Luca Bellucci, Matteo Agostini, Stefano Corni, Marco Cecchini, Giorgia Brancolini

**Affiliations:** ^1^ Dipartimento di Scienze Chimiche, Università di Padova, Padova, Italy; ^2^ Istituto Nanoscienze—CNR-NANO, Center S3, Modena, Italy; ^3^ NEST, Scuola Normale Superiore and Istituto Nanoscienze-CNR, Pisa, Italy

**Keywords:** acoustic wave biosensor, surface functionalization, molecular dynamics, Brownian dynamics, binding affinity, streptadivin-biotin assemblies

## Abstract

A rationally designed gold-functionalized surface capable of capturing a target protein is presented using the biotin–streptavidin pair as a proof-of-concept. We carried out multiscale simulations to shed light on the binding mechanism of streptavidin on four differently biotinylated surfaces. Brownian Dynamics simulations were used to reveal the preferred initial orientation of streptavidin over the surfaces, whereas classical molecular dynamics was used to refine the binding poses and to investigate the fundamental forces involved in binding, and the binding kinetics. We assessed the binding events and the stability of the streptavidin attachment through a quartz crystal microbalance with dissipation monitoring (QCM-D). The sensing element comprises of biotinylated polyethylene glycol chains grafted on the sensor’s gold surface *via* thiol-Au chemistry. Finally, we compared the results from experiments and simulations. We found that the confined biotin moieties can specifically capture streptavidin from the liquid phase and provide guidelines on how to exploit the microscopic parameters obtained from simulations to guide the design of further biosensors with enhanced sensitivity.

## Introduction

Biosensors with high affinity and selectivity require proper surface functionalization with selected bio-receptors. Bio-receptors are biologically derived molecules (e.g., proteins, peptides, or enzymes) that can selectively recognize and bind the analyte of interest. Self-assembled monolayers (SAMs) (Schwartz, 2001) enable tuning the surface properties (e.g., chemical reactivity, conductivity, and biocompatibility) of a given interface (Nicosia and Huskens, 2014). Among them, thiol-based SAMs (Singh et al., 2020) represent a convenient strategy to anchor bio-recognition elements on gold surfaces and nanostructures (Love et al., 2005; Wang et al., 2017), and also for sensing applications (Brothers et al., 2020). A robust and highly performing biosensor requires a careful setup of sensor functionalization (Wang et al., 2017) in terms of bio-receptor selection and a functionalization strategy. High specific binding should also be guaranteed, limiting the non-specific interferences from the background.

A well-known bio-recognition mechanism, already widely used in sensor-surface functionalization, is based on the biotin–streptavidin pair. This pair is characterized by the strong interaction between biotin and the tetravalent streptavidin (SA). Such interactions show high affinity (K_d_ ≈ 10^–14^ M^4^) and specificity. The literature studies report a number of experiments performed with biotinylated SAMs (b-SAMs), formed through thiol/metal (Pérez-Luna et al., 1999; Bacharouche et al., 2013) or silane/metal oxide chemistries (Singh et al., 2009). These features have made the SA–biotin pair widely used in biotechnology for labeling, detection, and purification (Wilchek and Bayer, 1988; Diamandis and Christopoulos, 1991). Despite the common use and the growing interest in SA/biotin technology, the understanding of the parameters that define the stability and orientation of SA on biotinylated surfaces is very limited (Migliorini et al., 2014; Osypova et al., 2015) since the corresponding orientation of SA on the surface can rarely be confirmed by experiments. Several macroscopic parameters such as chain length, charge of the head group of linker molecules are known (Lecot et al., 2020) to impact conformational fluctuations of Streptavidin while immobilizing over the surface and hence affecting biotin binding. Furthermore, a recent AFM and Steered Molecular Dynamics (SMD) study (Sedlak et al., 2020) highlighted the differences in an unbinding pathway with 4-fold different unbinding forces in pulling out biotin molecules from 4 different subunits of Streptavidin; in particular, owing to the tethered geometry of the linker molecule, the conformational changes of the biotin-binding loop of Streptavidin is affected differently for different monomeric units thus leading to potential discrepancy in the escaping mechanism of biotin. The existence of multiple energy barriers, followed by various intermediate states of biotin in the proximity of the binding pocket is observed over the course of the unbinding phenomenon as mentioned by the AFM experiment and SMD reports (Rico et al., 2019); the protein itself undergoes various induced fits with different kinetic rates in association with biotin, indicating the complex binding dynamics between Streptavidin and biotin.

Due to the experimental complexities to investigate self-assembled responsive devices for biosensing at the atomistic level, computational studies combining the *in silico* design and experimental measurements can be considered as an alternative approach to develop novel biosensors. The rational design of a number of functional components can be generally concertedly designed and arranged in a bottom-up approach, such as for self-assembled responsive devices for biosensing, drug delivery, and enzyme immobilization (Liu et al., 2007; Ogorzalek et al., 2015; Cholko et al., 2019; Puente-Santiago et al., 2019; Bolivar and Gallego, 2020; Singh et al., 2020; Watanabe et al., 2020).

A computational strategy for optimizing the functionalized surface density of an acoustic wave-based QCM sensor was introduced (Dutta et al., 2021) using polymeric chains of polyethylene glycol (PEG), supporting biotin moieties (bPEG) as the ligand unit for the SA analyte. Different biotinylation ratios between bPEG and non-biotinylated PEG were investigated by means of state-of-the art atomistic simulations showing how different microscopic parameters such as solvent-accessible surface area (SASA), distance distribution of biotin over the surface, radius of gyration of bPEG/PEG spacers, and ligand conformations can affect the availability of biotins for protein recognition.

In this article, we present a combined simulative and experimental study where atomistic simulations are explicitly including for the first time, the computed binding between four differently functionalized gold surfaces, and the SA protein tetramer, obtained by using different levels of theories [classical MD and Brownian dynamics (BD)] that cover multiple length- and time-scales. Brownian Dynamics rigid-body docking and fully atomistic MD simulations are sequentially combined to provide the molecular driving forces guiding the binding of SA to gold surfaces with a different degree of biotinylation. The results are analyzed and compared with the original experimental data, thus gaining insights into the protein–surface interactions. The role of different biotinylation densities, and of the flexibility of the polymeric chains in the protein–surface association process, is investigated.

The present work provides compelling evidence of the validity of the proposed approach combining multiple-level molecular calculations with experimental investigations. Additionally, the results provide information on the role of surface chemistry on the sensitivity of the biosensor pushing the knowledge in the field beyond the state-of-the-art technology.

## Results and discussion

### Docking of Streptavidin tetramer on (bPEG) n-linked gold surfaces

The possible adsorption orientations and the corresponding driving forces of the SA tetramer on gold surfaces having PEG surface densities of 0.4 molecule/nm (Nicosia and Huskens, 2014) and 0.8 molecule/nm (Nicosia and Huskens, 2014), respectively, are investigated to mimic the experimental (Agostini et al., 2019) surface density of PEG molecules on top of Au (111). For both surfaces, 100% of biotin-PEG (bPEG) as well as 50% of bPEG and 50% of PEG, are considered. Docking is performed with the protein–surface docking method implemented in SDA 7.2.2 (Martinez et al., 2015) by running Brownian dynamics simulations keeping the internal structure of the protein tetramer and the functionalized gold surface rigid. Initial conformation for the protein and the surfaces are obtained after 500 ns of MD simulations in explicit water (Mark and Nilsson, 2001; Kleinjung et al., 2012). The interaction (free) energy of the protein with the surface is obtained using an adapted version of the ProMetCS protein–metal force field (Kokh et al., 2010), and adsorption free energies of SA tetramer on the (bPEG) n-linked gold surfaces are computed for the structures resulting from the docking. The protein surface encounter complexes are obtained from the BD simulation and the trajectories are clustered to identify significantly different protein orientations. For each of the most populated complexes, ranked by size, a representative structure is selected for each system as the initial starting structure for MD refinement.

**FIGURE 1:**
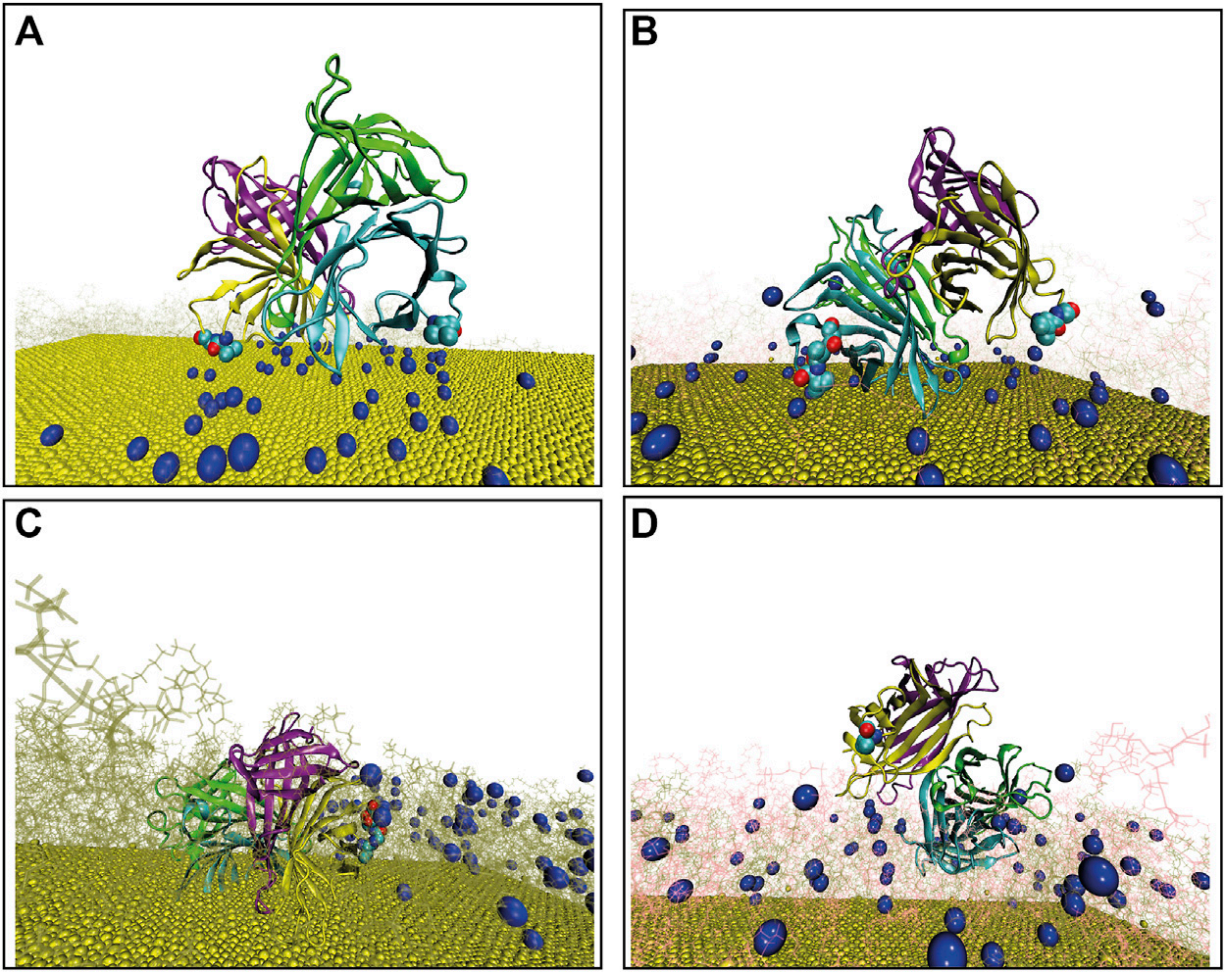
Final configuration of Streptavidin as docked over four different surfaces, terminal biotin atoms are shown by spherical representation (blue), Au (111) in yellow, PEGs are in pink, bPEGs in green, and the protein is shown in a cartoon representation with four different colors for tetramers, vdW representation of proteins are the residues in proximity with bPEG/PEG in the docked complex; **(A)** 49bPEG; **(B)** 25bPEG-24PEG; **(C)** 98bPEG; and **(D)** bPEG49-peg49.

When this docking procedure is applied to the four-surface systems denoted as i) 49bPEG, ii) 25bPEG-24PEG, iii) 98bPEG, and iv) 49bPEG-49PEG, respectively, it yields five different orientations accounting for 100% of the encounter complexes obtained, see Table 1. The representative structure of each computed complex is shown in Figure 1.

**TABLE 1:** Au (111) with different functionalizations docked with Streptavidin, results as obtained from SDA.

System	Clust index	RelPop (%)^a^	$\left(U_{Repr}\right)$ kJ/mol^b^	$\left(U_{EP}\right)$ kJ/mol^c^	$\left(U_{ds}^{e}\right)$ kJ/mol^d^	$\left(U_{ds}^{h}\right)$ kJ/mol^e^
49bPEG	A1	36.6	− 64.16	− 4.49	12.10	− 71.80
**49bPEG**	**A2**	**36.6**	− **65.82**	**3.25**	**13.57**	− **82.73**
49bPEG	A3	13.8	− 62.11	3.62	8.67	− 74.49
49bPEG	A4	10.6	− 63.39	− 0.50	12.57	− 75.54
49bPEG	A5	2.4	− 63.62	− 0.94	5.76	− 68.51
						
25bPEG-24PEG	B1	24.8	− 72.00	− 1.44	9.17	− 79.81
**25bPEG-24PEG**	**B2**	**30.7**	− **66.12**	− **2.05**	**12.35**	− **76.42**
25bPEG-24PEG	B3	26.6	− 63.14	− 1.99	14.67	− 75.90
25bPEG-24PEG	B4	14.2	− 64.24	− 1.13	8.17	− 71.36
25bPEG-24PEG	B5	3.7	− 64.11	− 1.69	3.16	− 65.65
						
98bPEG	C1	17.7	− 41.25	− 8.51	10.31	− 43.10
**98bPEG**	**C2**	**62.5**	− **41.69**	− **2.80**	**4.79**	− **43.72**
98bPEG	C3	16.4	− 40.98	− 4.96	4.42	− 40.49
98bPEG	C4	1.8	− 41.01	− 1.32	4.40	− 44.09
98bPEG	C5	1.6	− 42.28	− 1.97	3.65	− 44.01
						
**49bPEG-49PEG**	**D1**	**46.6**	− **49.49**	− **3.65**	**4.77**	− **50.67**
49bPEG-49PEG	D2	19.4	− 48.67	− 0.52	2.73	− 50.94
49bPEG-49PEG	D3	15.3	− 51.90	1.45	7.69	− 61.10
49bPEG-49PEG	D4	10.4	− 50.04	−4.37	3.57	− 40.06
49bPEG-49PEG	D5	8.3	47.76	− 1.91	3.09	− 49.00

^a^ Relative population of this cluster.

^b^ $U_{Repr:}$ Total interaction energy of the representative cluster in kJ/mol.

^c^ $U_{EP}$ : total electrostatic energy of the representative complex, in kJ/mol.

^d^ $U_{ds}^{e}$ : electrostatic desolvation energy of the representative complex, in kJ/mol.

^e^ $U_{ds}^{h}$ : hydrophobic desolvation energy of the representative complex, in kJ/mol. The docking orientation that is being chosen for MD refinement is represented in bold format.

Bold values indicated the docked structures which were selected as initial input for the MD simulations.

During docking, the interaction energy between the protein and the surface $\left(U_{Repr}\right)$ is described by three main terms: (a) long-range electrostatic interactions $\left(U_{EP}\right)$ , (b) short-range electrostatic desolvation of the protein $\left(U_{ds}^{e}\right)$ , and (c) non-polar desolvation i.e., equivalent to hydrophobicity-mediated interaction $\left(U_{ds}^{h}\right)$ between the binding macro-molecules. Details of this calculation can be found in the Supporting Information (SI). The detailed binding energetics for our systems is shown in Table 1. In bold, the resulting encounter complexes of the most populated and energetically stable clusters, namely A2, B2, C2, and D1, are highlighted since they are chosen as the initial starting structures for the MD simulations.

The representative structure of each computed complex is shown in Figures 1A–D. Binding in complexes A, B, C, and D is driven mostly by hydrophobic desolvation ( $U_{ds}^{h}$ ) interactions, but in complexes C and D, the electrostatic terms contribute more favorably to binding. The difference between the binding energies in complexes A and B comes from a slightly more favorable electrostatic energy for complexes B. The difference between complexes C and D comes from a more favorable hydrophobic energy for complexes D. Overall, the encounter complexes on 50% of bPEG and 50% of the PEG surface have always higher binding energies with respect to 100% of the bPEG counterpart, both at low density (A and B) and at high density (C and D). Next, we analyze the residues belonging to the biotin-binding loop (Le Trong et al., 2011) of tetrameric Streptavidin that come into the proximity of ligands bPEG/PEG after docking; we impose a distance threshold ∼5 Å to identify such residues. The strongest binding seems to be associated with the total amount of residues contacting the surface with a small preference for non-polar amino acids e.g., VAL and SER with respect to other aliphatic residues of SA-binding pockets. From the present docking results, we may conclude that for bPEG49 (Figure 1A; Supplementary Figure S1A), two contacts are used, namely VAL47 of chain B and VAL47, GLY48 of chain D which are participating in the binding and contribute to the interaction energy of complex A2 ∼ −65.82 kJ/mol. For bPEG25-PEG24, many contacts are used in order to optimize the binding energy of complex B2 ∼ −66.12 kJ/mol, including SER45, ALA46, and VAL47 of chain B and ALA46, VAL47, GLY48, and ASN49 of chain D (Figure 1B; Supplementary Figure S1B). Therefore, in mixed functionalization, the protein seems to be able to form a higher number of contacts. Thus in the case of encounter complexes B on 25bPEG-24PEG, the binding energy is slightly more stable than in 49bPEG. As the grafted linker density increases, the docking positions referred to as C and D encounter complexes involve only a single binding loop of the tetrameric SA, and this is because the protein is rarely able to simultaneously form contacts with two binding loops. In the case of 98bPEG, the residues are VAL47, GLY48, ASN49, and ASP50 of chain D of SA (Figure 1C; Supplementary Figure S1C) resulting in complex C2 with the binding energy ∼ −41.69 kJ/mol. In the case of bPEG49-PEG49, the residue is VAL47 of chain D of SA (Figure 1D; Supplementary Figure S1D) resulting in D2 ∼ −49.49 kJ/mol. The latter results reflect the fact that the population and stability of those orientations can be affected more by conformational relaxation of the protein and of the surface during MD refinement compared to the lower density surfaces, as it will be discussed in the next section.

To summarize, the docking results provide an overview of the protein contacting residues that are close to the surface in the early protein/surface binding from which we may conclude that SA on low-linker density and high-linker density surfaces, can make two types of bound complexes: A, B, in which many contacts can be used in order to optimize the binding energy, and C and D, in which a small contact area is compensated by electrostatic interactions. Docking positions C and D appear to have a lower binding energy with respect to A and B, in the rigid docking results, and this is because the protein is rarely able to simultaneously form contacts with more than one binding loop in the absence of structural relaxation. This is mainly due to the limitation of the rigid docking procedure which mimics the functionalized gold surface as a rigid surface. As it will be reported in the next section, the final binding orientations and the underlying microscopic phenomena occurring at the protein/surface interface are further clarified running fully flexible MD simulations.

### Refinement of protein–surface encounter complexes by MD simulations

To investigate the binding and stability of the most populated and most stable docked encounter complexes for each surface, the changes in protein structure/orientation upon adsorption are followed performing 500 ns MD simulations starting with the most representative cluster for each system, namely A2, B2, C2, and D1 complexes obtained from rigid-body BD docking. The simulations are based on the GolP (Iori et al., 2009) force field with the SPC/E (Mark and Nilsson, 2001) water model as implemented in the GROMACS (Van Der Spoel et al., 2005; Hess et al., 2008). Before the addition of the water molecules, the center of mass of the protein was placed at 50 Å from the gold surface (the averaged thickness of the functionalized layer is lying at ∼25 Å) as shown in Supplementary Figures S2A–B, but retaining the original docked orientation with respect to the surface, in order to avoid kinetics traps.

**FIGURE 2:**
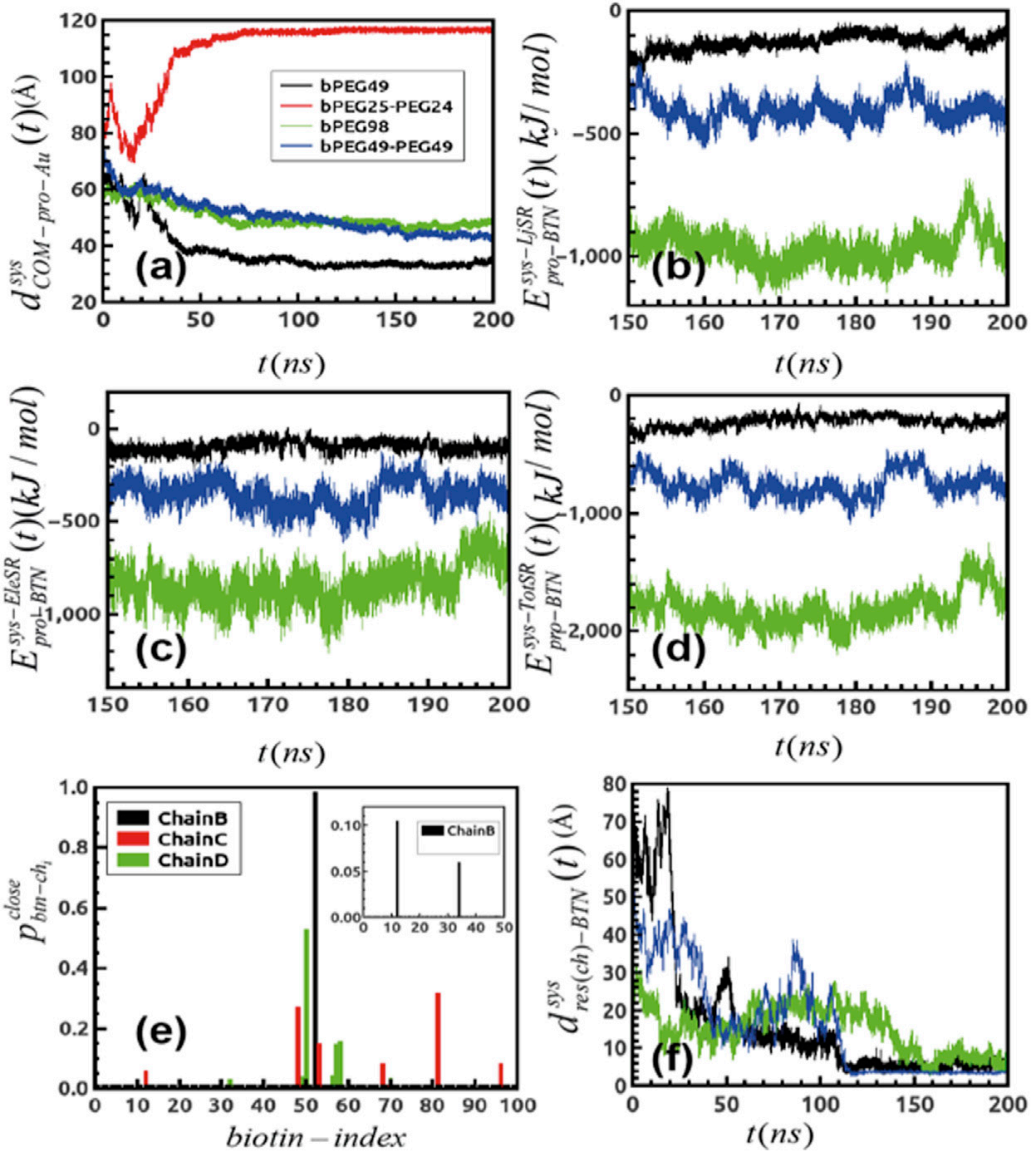
**(A)** Distance fluctuation between the center of mass of protein over the gold surface for differently functionalized surfaces. **(B)** Short-ranged LJ interaction between protein and all the biotins generated over trajectories and that for **(C)** short-ranged electrostatic components **(D)** and finally, the total additive binding energetics for the systems; black: bPEG49, green: bPEG98, and blue: bPEG49-PEG49. **(E)** Probability of finding biotins close to binding loops of Streptavidin for different chains of bPEG98 and same for bPEg49-Peg49. **(F)** Distance fluctuation between the terminal biotin atom and the residue of Streptavidin forming binding over MD. The color code is same as in Figures 2B−D.

We first verified whether the simulations converged, by means of root mean squared deviations, and RMSD (details in SI), evaluated with respect to protein atoms as well as for ligands atoms, as reported in Supplementary Figures S2A–F. To identify the adsorption of SA to the differently functionalized surfaces, we calculated the distance fluctuation over time between the z coordinate of the center of mass of the protein to the gold surface $\left(d_{COM-pro-Au}^{sys}\left(t\right)\right)$ , as reported in Figure 2A. In case of the bPEG49 surface $d_{COM-pro-Au}^{bPEG49}\left(t\right)$ , the protein is initially only slowly attracted toward the surface before initiating binding, but once the binding is established, namely after the first 40 ns–50 ns, it is stabilized and maintained over the last 100 ns of the trajectories, with a center-to-center distance of ∼35 Å (black in Figure 2A). On the contrary, for the mixed-functionalized bPEG25-PEG24 surface, the protein is initially attracted toward the surface, but after the first 20 ns, the protein is able to detach from the surface, drifting away from it, as shown by the values of distance $d_{COM-pro-Au}^{bPEG25-PEG24}\left(t\right)$ ∼ 130 Å (red in Figure 2A). This indicates that MD refinement highlights the differences in binding of SA on bPEG49 and bPEG25-PEG24 due to different surface coverage and biotin content, thus showing how different densities of biotin can induce different stabilities of initial binding poses. The result suggests that at the low-density coverage, the stability of SA binding is affected by the biotinylation ratio, with an induced detachment when reducing the biotin fractions of 50%. Thus, a minimum number of biotins are required, especially for a “fluid” surface in which ligands are more flexible, due to the low density of surface coverage. Conversely, at high-density coverage, namely for $d_{COM-pro-Au}^{bPEG98}\left(t\right)$ and $d_{COM-pro-Au}^{bPEG49-PEG49}\left(t\right)$ , the protein is able to form stable bindings toward the surface, almost independently by the biotinylation ratio. In both cases, the binding is maintained over time and the center-to-center distance is settled around ∼55 Å. This reflects the different behaviors based on the surface-packing density of ligands and it can most likely be explained as the enhanced ability of SA to be attracted and to rearrange toward more rigid and high-density surfaces, with respect to a more fluid surface.

The comparative MD study enables establishing the guiding interactions through an energy decomposition analysis. The protein–surface interaction energy components are given in Figures 2B–D through an energy decomposition analysis of the intermolecular van der Waals and Coulomb interactions, which are computed using the rerun gromacs option on the extracted frames.

The interactions are computed between all atoms of SA and all atoms of biotin, by extracting short-ranged Lennard Jones (LJ), namely $E_{pro-BTN}^{sys-LjSR}\;\left(t\right)$ in Figure 2B, and short-ranged Coulombic components, namely $E_{pro-BTN}^{sys-EleSR}\;\left(t\right)$ in Figure 2C. The overall additive energetics is referred to as $E_{pro-BTN}^{sys-TotSR}\;\left(t\right)$ in Figure 2D. The results indicate that binding interaction energy of the bPEG98 surface has a higher net contribution of both by LJ and Coulombic interactions with respect to the other surfaces; the binding interaction strength of bPEG49-PEG49 is driven both by LJ and Coulombic interactions but at a smaller extent with respect to the corresponding being fully biotinylated. For the low-density surface, bPEG49, the binding is driven mainly by Coulombic interactions. As a consequence, the overall direct interaction energy is stronger for bPEG98 with ∼ −1800 kJ/mol, followed by bPEG49-PEG49 ∼ −600 kJ/mol and finally, least in bPEG49 ∼ −200 kJ/mol. Thus, the larger the number of biotins, the stronger the direct interaction with the SA protein; however, for an equal number of biotins, the mixed-functionalized surface is able to provide a stronger binding interaction, confirming the ability of the protein to better rearrange and stabilize on a more dense and rigid surface.

To better assess the specific SA–biotin association binding dynamics in the case of the most favorable surfaces, namely bPEG98, bPEG49-PEG49, and bPEG49, the probability of finding a biotin at distances within 5 Å to each of the four biotin binding loops of SA tetramer was extracted from the simulated trajectories $\left(p_{btn-{ch}_{i}}^{close}\right)$ . Figure 2E reports the presence of biotins belonging to the bPEG98 surface in the vicinity of three different biotin-binding loops of SA, namely chains B, C, and D, during different intervals of time of the entire MD simulation. From the present refinement result, we may conclude that a favorable orientation of the SA tetramer allowing three different binding sites over four to be in contact with exposed biotin molecules from the surface is used in order to optimize the binding. It can be assumed that the binding in this case becomes stronger as a result of a cumulative effect of the protein–biotin interactions. Conversely, in the presence of the bPEG49-PEG49 surface, the protein is able to orient only one binding site toward the surface, as reported in the inset of Figure 2E, thus providing a contact with only two biotin molecules. The interaction with the bPEG49 surface results in an even weaker orientation of the SA tetramer contacting only one biotin over a significantly shorter period of time (plot is omitted due to very small magnitude of $p_{btn-{ch}_{i}}^{close}$ ).

In Figure 2F, the distance fluctuation plots are reported, showing the variation of the distance between the contacting residues of SA and the terminal atom of the biotin as a function of simulation time ( $d_{res\left(ch\right)-BTN}^{sys}\left(t\right)$ ). Some sample cases are shown in Figure 2F, for both $d_{A50\left(B\right)-BTN}^{bPEG49}\left(t\right)$ and $d_{A46\left(D\right)-BTN}^{bPEG49-PEG49}\left(t\right)$ , the residues involved in the binding are ALA50 of chain B and ALA46 of chain D, forming stable contacts with biotins. Initially, the distance is larger ∼70 Å for the bPEG49 surface and smaller ∼45 Å for the bPEG49-PEG49 surface, but in the last ∼80 ns time period of simulation both distances settled at ∼5 Å. However, for $d_{A50\left(C\right)-BTN}^{bPEG98}\left(t\right)$ , the interaction is observed to be stronger even at initial time steps, as shown by ∼30 Å of distance.

**FIGURE 3:**
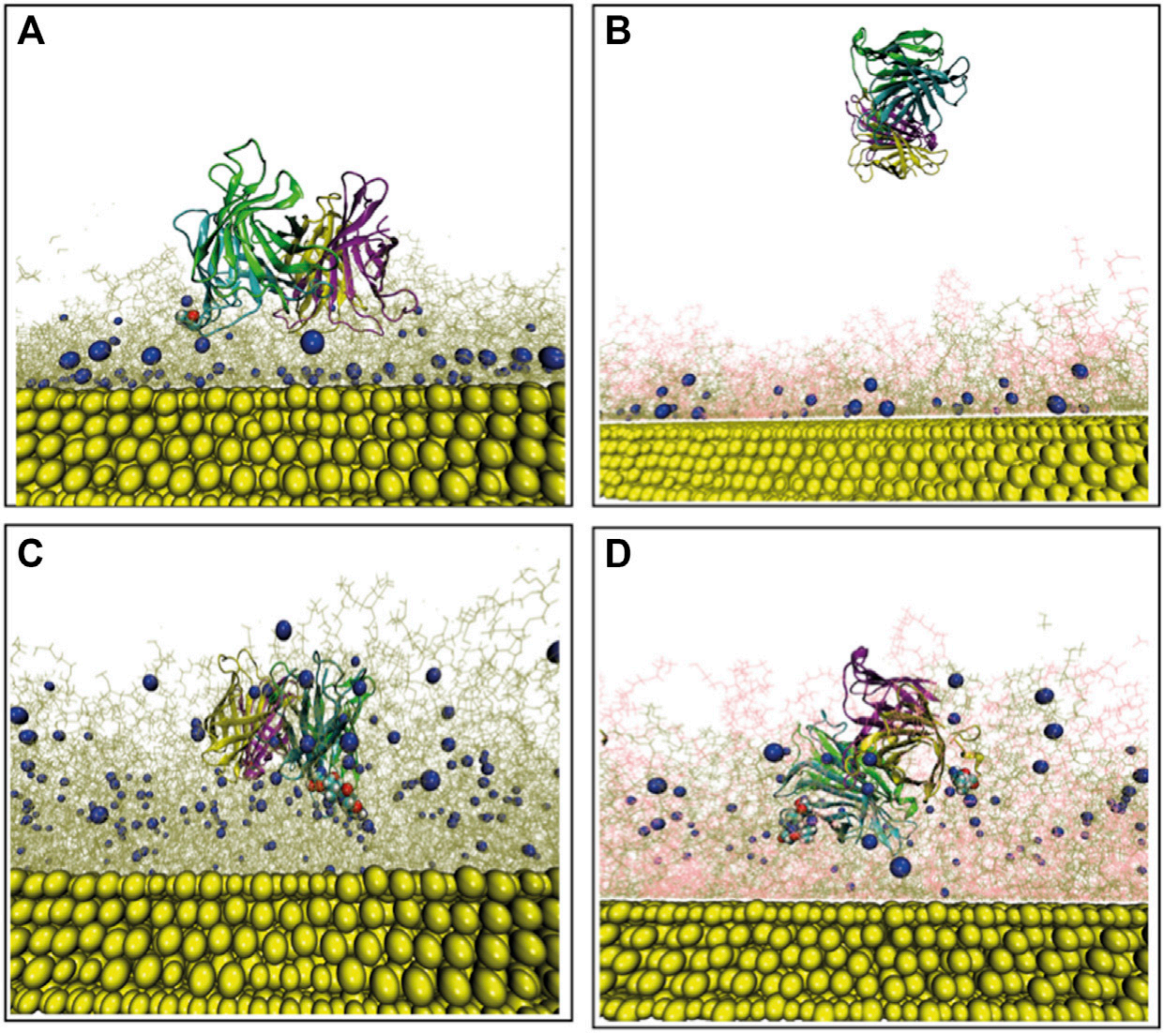
Final configuration of Streptavidin over four different surfaces as obtained after MD simulation and represented from the top as well as side views, terminal biotin atoms are shown by spherical representation (blue), Au (111) in yellow, PEGs are in pink, bPEGs in green, and the protein is shown in a cartoon representation with four different colors for tetramers, vdW spheres of proteins are the residues in proximity with bPEG/PEG in docked complex; **(A)** bPEG49; **(B)** bPEG25-PEG24; **(C)** bPEG98; **(D)** bPEG49-peg49.

In order to acquire a better understanding of the role of amino acids having the highest binding affinities for biotin to the overall SA-surface binding, specific SA amino-acids belonging to the biotin-binding loop (Le Trong et al., 2011) (i.e., residue index 45 to 52 in each monomer) are analyzed using the resulting final frame of the protein–surface binding configurations (after 500 ns MD). The protein residues contacting biotin (terminal) atoms at distances <5 Å are identified. Figure 3A represent the contacting SA residues (red balls) and the biotin terminal atoms (blue balls) for the low-density bPEG49 surface. More specifically, the non-polar residue ALA50 belonging to chain B of SA is found at distances closer than < 5 Å to one particular biotin, signifying the signature of interaction, as also clearly represented in Supplementary Figure S4A. The final configurations of SA on bPEG25-PEG24 surface are also depicted in Figure 3B, but due to detachment of the SA during the MD simulation, at the end of 500 ns of MD simulations, no protein residue is found to be in contact at short distances. Thus, the bPEG25-PEG24 surface is no longer considered as a valuable choice in the design of the functionalization of the biosensor, and it is excluded from further analysis. The strongest binding is found for bPEG98, here SER45, ALA46, VAL47, GLY48, and ASN49 of chain B make contact with two such biotins, followed by ALA50 of chain C towards a third biotin as depicted in Figure 3C, followed by Supplementary Figure S4C. Conversely, for the high density bPEG49-PEG49 surface, the protein-surface contact area is found to be enhanced with respect to mixed low density surface (Figure 3B). Specifically, residues SER45, ALA46, VAL47, and GLY48 of chain B along with ALA46, GLY47 of chain D are found within 5 Å of two particular biotins (Figure 3D, also in Supplementary Figure S4B).

Overall, the MD simulations are suggesting the bPEG98 surface as a suitable choice for a robust detection of SA, and conversely, that a monovalent SA anchoring to the surface is not stable and may lead to the detachment of the protein from the surface. Simulations show that more dense and rigid surfaces even with moderately biotin content can be optimal for such biosensor architectures.

As a result of the structural changes induced on the functionalized gold surface upon protein adsorption, we do expect the solvent exposure of biotins to change with respect to the case in which the SA protein was absent. We computed the Solvent-Accessible Surface Area (SASA) of biotins (details in SI) (Mark and Nilsson, 2001) and we compared the data with the SASA of functionalized gold surfaces without the protein.

The SASA for each single biotin was computed over the simulation trajectories and the values were grouped in three different regimes, corresponding to the different degrees of solvent exposure. It is estimated that the SASA for a fully solvated biotin is 4 nm^2^, thus the SASA of each biotin belonging to the investigated surfaces is qualitatively classified in three regimes, the first having SASA of biotins <0.25 nm^2^, the second 0.25 nm^2^ < SASA of biotins *>* 2.1 nm^2^, and the third more having SASA of biotins >2.1 nm^2^, as reported in Table 2. The analysis of the SASA of biotins in the absence and in the presence of SA binding reveals that the exposure of the biotin molecules composing the functionalization is significantly changed by the introduction of protein. By investigating the differences in the SASA values of the biotin molecules before [Table 1 in ref (Dutta et al., 2021)] and after the binding of SA (Table 2), the SA binding slightly increases the exposure of biotins belonging to the second and the third regimes of exposure, respectively, from 53.9% to 63.2% and from 1.2% to 2.8% for bPEG49-peg49; from 45.1% to 52.8% and from 1.2% to 3.2% in the 98bPEG case. Conversely, the SASA of biotins decreases from 31.9% to 19.7% and 1.7%–1.6% in the case of low-density surface bPEG49 since the majority of biotins remain buried under the footprint of proteins.

**TABLE 2:** Three different regimes of SASA of biotins generated for different surfaces over trajectories.

System	SASA of biotins <0.25 nm^2^ (%)	SASA of biotins > 0.25 & < 2.1 nm^2^ (%)	SASA of biotins 2.1 nm (Nicosia and Huskens, 2014)
9 (%)8bPEG	44.0	52.8	3.2
49bPEG-49PEG	34.0	63.2	2.8
49bPEG	78.7	19.7	1.6

Since the overall SASA of biotins belonging to the second and third groups is found to be larger for the bPEG49-PEG49 with respect to 98bPEG, for a fully biotinylated high-density surface, the saturation is expected to come earlier than in its half biotinylated counterpart.

## Comparison with experimental results

### Functionalization with PEG-based layers

**FIGURE 4:**
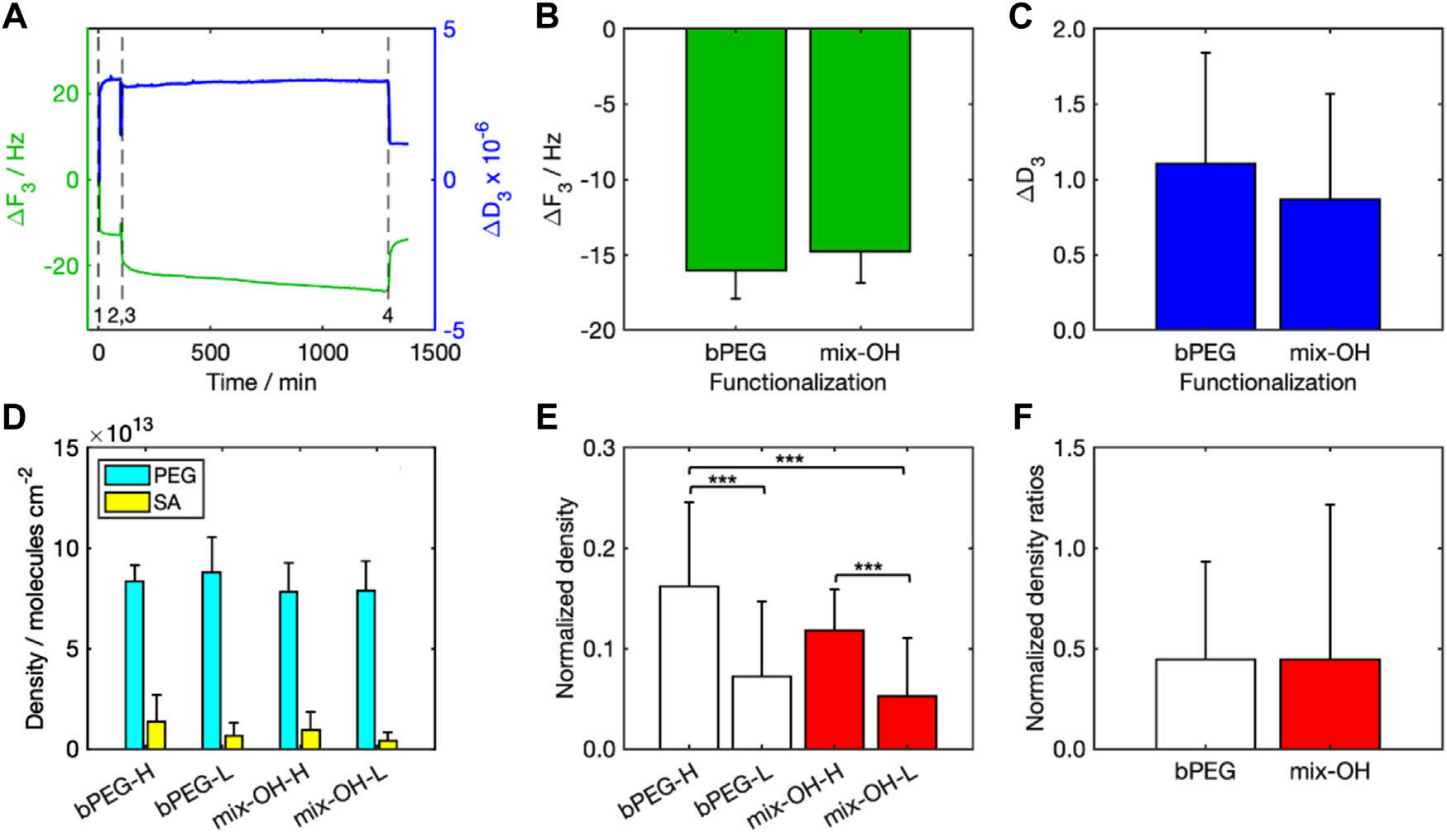
Results of QCM-D analysis: **(A)** ΔF3 and ΔD3 over time (event 1: PEG injection, event 2: PEG rinsing, event 3: SA injection, event 4: SA rinsing), **(B)** values of ΔF3, and **(C)** ΔD3 (after rinsing) for bPEG and mix-OH functionalization, **(D)** density of molecules per cm^2^ calculated for the functionalization layer and bound SA in all experiments, **(E)** normalized density calculated for both functionalizations, bPEG- and mix-OH indicate the functionalization layer while letters H and L indicate the concentration of SA in the sample. The symbol *** indicates a statistical significance with p < 0.001 (Tukey–Kramer test), **(F)** ratios between the normalized densities calculated for both SA concentrations.

Figure 4A shows one characteristic sensorgram acquired by the QCM-D representative for a complete experiment (Figure 4A). A reduction in the crystal resonance frequency (*Δf* < 0) was measured during both PEG functionalization (0 min, event 1) and SA detection (75 min, event 3), followed by a slight increase after the rinsing steps (70 min, event 2, and 975 min, event 4). Frequency and dissipation are inversely proportional (Duner et al., 2013), thus the trends were opposite. *Δf* after rinsing was comparable between the two tested functionalizations (bPEG and mix-OH, Figure 4B). The values of *ΔD* after rinsing (Figure 4C) were smaller than the taken reference value for the application of the Sauerbrey model (see the Methodology section).

### Streptavidin detection

Molecular densities over the sensor surface calculated for PEG and SA (Figure 4D) were significantly higher for PEG than SA, indicating that the number of PEG molecules attached to the sensor surface was about 6–8 times and 14–19 times larger than the number of SA molecules immobilized over the functionalization layer, for experiments at high and low SA concentrations, respectively.

To take into account the effect of the functionalization variability, the molecular density after rinsing obtained with SA was normalized to those obtained after the relative functionalizations (Figure 4E). The mean values highlighted significant differences between the experiments by changing the SA concentrations for both functionalizations. On the other hand, the differences between the fully biotinylated and the mixed functionalizations are not significant. The calculated normalized density ratios were around 0.45 (Figure 4F). Since the amount of SA molecules is ten times lower in the SA-L experiments than in SA-H ones, normalized density values in SA-L experiments were significantly higher than expected. It indicated a potential saturation of the biotin moieties in the SA-H experiments.

The results are in agreement with the atomistic simulations pointing to the higher surface density as most favorable to the SA binding associated to a larger exposure of biotins to SA. The experimental data are further supported by binding kinetics calculations, see Supplementary Figure S5A–C, demonstrating that the details of the binding interface have an appreciable effect on the stability of the SA attachment. Binding affinity is quantified by kinetic rate simulations and it is clear from the data (Supplementary Figure S5B) that the average residence lifetime of SA binding on 25bPEG-24PEG is drastically reduced compared to 98bPEG, while it is similar for both 49bPEG-49PEG and 49bPEG having the same number of biotins. The results show that the slowest binding dissociation rates (k_off_) (or the largest residence times) are occurring at the highest surface coverage, namely 98bPEG (Supplementary Figure S5C). Remarkably, both experiments and simulations indicate a potential faster saturation of the SA-H systems with respect to SA-L, as discussed in the previous section.

## Conclusion

We have integrated the computational and experimental designs of a gold-based functionalized biosensor capable of capturing a target protein. The sensing elements comprise biotin molecules immobilized on thiolated PEG chains grafted on the gold surface, designed to target an arbitrarily binding site on the surface of the SA tetramer. We have used atomistic simulations at multiple levels of theory, combining docking by Brownian dynamics and kinetics and classical atomistic MD with a state-of-the-art force field. From these simulations, we have provided molecular insights into the stability and the orientation of SA binding to four differently biotinylated surfaces, providing information not directly accessible from experiments alone. In particular, on the basis of our results obtained at various biotinylation ratios and at different surface coverages during SA surface docking, we discussed the nature of the interactions that guide the binding of SA to different surfaces, finding that short-range, non-electrostatic interactions can be the leading factors for the initial encounter complexes.

Remarkably, the refinement with atomistic simulations indicates that the measured higher quantity of the SA protein deposited on the high-density surface is in agreement with the calculated data and it stems from a direct interaction of SA residues with the exposed biotins on the surface (e.g., on bPEG98) which are instead buried under the footprint of the protein in the low-density surface (e.g., bPEG49). Thus, SA binds more favorably on rigid high-coverage density surfaces with respect to flexible low-coverage density surfaces. Finally, both experiments and simulations suggest that a more rapid saturation is expected to occur at the higher density surface coverage.

Our results show that the functionalized biotins are capable of specifically capturing SA from the surface–liquid interface with micro-molar affinity. Here, the SA–biotin pair has been used as a proof-of-concept in view of a further challenge, namely the design of more complex devices in the form of coupled binders (Dutta et al., 2022) or antibody fragments (Yang and Shah, 2020) in the quest for biosensors with enhanced binding affinity.

## Materials and methods

### Protein-surface docking

We considered a grid of dimensions 161 × 161 × 161 Å, a grid-spacing of 1.0 Å to build the electrostatic potential grid using APBS program (Baker et al., 2001). A salt concentration of 10 mM was included as a non-specific screening effect on the electrostatic potential of the protein which was calculated using the APBS program. All titratable protein side chains were assigned their standard protonation states at pH 7.0 with H++ (Anandakrishnan et al., 2012), corresponding to the experimental pH. Simulations were performed at room temperature ∼300 K. The initial structures for the four surfaces and for the SA protein analyte were obtained after 500 ns MD simulations. For every system, 5,000 runs each with 500 ns of simulation time were performed to obtain the preferred binding orientation on the surface over trajectories, whereas the position and orientation of the protein was updated following the Metropolis algorithm during BD. The simulation was performed within a box having spherical symmetry, the beginning center of protein is placed at 100 Å distance from the center of the surface. 1 ps simulation time step was maintained as long as the center-to-center distance remains < 50 Å. For the fully biotinylated surfaces, terminals N1, N2, and O3 atoms of biotins are considered to calculate effective charges, these atoms also play (Le Trong et al., 2011; Song et al., 2015) significant roles in forming H bonds with the biotin-binding loops of Streptavidin, whereas for the half biotinylated cases, terminal O atoms of non-biotinylated PEGs were also included. For the protein, N atoms were chosen for basic residues and O for the acidic ones. The translational and rotational diffusion coefficients for the larger density surfaces i.e., for 98 bPEG and 49bPEG-49PEG were found ∼0.002 cm^2^/s and 0.35 $\times 10^{-6}$ rad (Nicosia and Huskens, 2014)/ps, respectively, as per the HYDROPRO (Ortega et al., 2011) software. Similarly, for smaller density surfaces, translational and rotational diffusion coefficients were ∼0.004 cm^2^/s and 0.70 $\times 10^{-6}$ rad (Nicosia and Huskens, 2014)/ps. For Streptavidin, 0.0073 cm^2^/s and 0.60 $\times 10^{-5}$ rad (Nicosia and Huskens, 2014)/ps were used. The 1,000 lowest energy configurations were saved, a threshold root mean square deviation (RMSD) 1 Å was used to distinguish between two different docking positions of protein over the gold surface.

### Molecular dynamics refinement

Topology and forcefield for PEG and bPEG were developed in-house (Dutta et al., 2021). Those for Au (111) surface atoms were obtained from the GolP all-atom classical force field (Iori et al., 2009; Hoefling et al., 2010; Brancolini et al., 2015; Cantarutti et al., 2017; Brancolini et al., 2018; Brancolini et al., 2019), and polarization of the gold surface atoms is particularly taken into account. The covalent bond length between (AU) and the S atom of spacers (bPEG/PEG) were restrained, while the AU-S-C angle and dihedral AU-S-C-C were free to rotate during the simulations. Bonded and non-bonded LJ FF parameters were developed *ad hoc* for the systems (Bizzarri et al., 2003). 200 ns MD^46-47^ were performed in a water-box with OPLS and an explicit SPC/E (Mark and Nilsson, 2001) water model using GROMACS (Pronk et al., 2013). We used a rectangular box of dimensions 10.7 × 10.3 × 15 nm for all the four cases and ∼150,000 numbers of atoms are included. We implement periodic boundary condition (PBC) (Allen and Tildesley, 1989; Frenkel and Smit, 2002), steepest descent algorithm (Mcsherry, 1976) was used to minimize the systems to 50,000 steps. The leap-frog algorithm with a time step of 2 fs was considered The temperature at 300 K in terms of V-rescale-modified Berendsen thermostat (Bussi et al., 2007) and pressure coupling (1 bar) through the Parrinello–Rahman barostat (Bussi et al., 2007) were achieved. NVT (isothermal isochoric) and NPT (isothermal isobaric) equilibrations for 100 pico second (ps) were performed. Short-range cut-off for van der Waals and for electrostatic interactions ∼1.2 nm were applied. The Particle Mesh Ewald (PME) method was used (Essmann et al., 1995) for long-ranged electrostatic interactions. LINCS algorithm, used to constraint bond length and Maxwell Boltzmann distribution at a prescribed temperature, was used to assign velocities. Trajectories were saved in every 2 ps. All the analyses were performed on equilibrated isothermal isobaric trajectories.

### Quartz crystal microbalance with dissipation monitoring experiments

All the QCM-D (E4 model, Q-Sense AB, Sweden) measurements were performed with polished AT-cut quartz crystals (gold electrodes, fundamental resonance frequency *f0* = 5 MHz, diameter = 14 mm, and thickness = 100 nm) in the static mode (stop flow). The fluidic cells were maintained using a thermostat at 25°C. This apparatus allowed recording the crystal resonance frequency shift (*Δf*) and energy dissipation (*ΔD*) simultaneously for up to 13 overtones, by exciting the fundamental resonance frequency of the crystal. In this work, we have chosen the data analysis of the 3rd overtone as the most sensitive and stable among the entire dataset. *ΔD* values, which are strictly related to the mechanical behavior of the functionalization ad layers, were checked for the application of the Sauerbrey model. In this work, criterium1 used to consider the Sauerbrey model valid is *ΔD* < 2.0 × 10^6^.

### Sensor surface functionalization

Heterobifunctional thiol-polyethyleneglycol-biotin (bPEG, Mw 2 kDa, NANOCS Inc.) and thiol-polyethyleneglycol-hydroxyl (PEG-OH, Mw 2 kDa, NANOCS Inc.) were used for quartz functionalization as binding molecules. PEG derivatives were dissolved in water (molecular biology purity degree, Merck). We prepared a bPEG solution with concentration 2 mg ml^−1^, and a mix solution (mix-OH) containing both bPEG and PEG-OH at a 1:1 molar ratio and an overall PEG concentration of 2 mg ml^−1^. Prior to use, the crystals were treated with plasma oxygen (Femto Diener, 10 min, power 100 W), washed with a 5:1:1 solution of water, ammonia (32% v/v), and oxygen peroxide (25% v/v) at 75°C for 15 min, rinsed with water and then with isopropanol, finally treated with plasma oxygen again (10 min, power 100 W). The crystal quartz gold surface was modified by covalently bonding thiolated PEGs *via* thiol-gold chemistry. To this end, sensors were first rinsed with water and stabilized waiting for a sufficient time until the acquired signals did not show drifts (ΔF < 1 Hz over 30 min). Then, a bPEG or a mix-OH solution was injected in the flow cell. We let the solutions in contact with the gold surfaces for 70 min before rinsing with water for 5 min.

### Detection experiments

Streptavidin (SA, Mw 53 kDa, IBA LifeScience) was chosen as the protein to be bound to the functionalized quartz crystal sensors. SA was dissolved in a phosphate buffer solution (PBS, Merck) at two concentrations, indicated in the text as high (H, 0.1 mg ml^−1^) and low (L, 0.01 mg ml^−1^). After functionalization, a SA-H or SA-L solution was injected in the QCM-D flow cells and data were acquired for 900 min in a static condition. Finally, the sensors were rinsed with water and data were acquired for further 170 min.

## Data Availability

The raw data supporting the conclusion of this article will be made available by the authors, without undue reservation.
