# Supplementary material for: Tuning gold-based surface functionalization for streptavidin detection: A combined simulative and experimental study
Source: Front Mol Biosci. 2022 Nov 28;9:1006525. doi: 10.3389/fmolb.2022.1006525 (PMC9742443; doi:10.3389/fmolb.2022.1006525)
Supplement: Supplementary file 1 [file DataSheet1.PDF]

SUPPORTING INFORMATION

"Tuning Gold-Based Surface Functionalization for Streptavidin Detection: a Combined Simulative and Experimental Study"

Sutapa Dutta[1,3], Mariacristina Gagliardi[2], Luca Bellucci[2], Matteo Agostini[2], Stefano Corni[1,3], Marco Cecchini[2], Giorgia Brancolini[3*]*

[1] Dipartimento di Scienze Chimiche, Università di Padova, 35131 Padova, Italy

[2] NEST, Scuola Normale Superiore and Istituto Nanoscienze-CNR, Piazza San Silvestro 12, 56127 Pisa, Italy

[3] Istituto Nanoscienze – CNR-NANO, Center S3, via G. Campi 213/A, 41125 Modena, Italy

Corresponding author e-mail: giorgia.brancolini@nano.cnr.it, marco.cecchini@nano.cnr.it

Details of docking calculation:

In order to make the calculation of electrostatic potential computationally less expensive, an effective charge model (ECM) is incorporated in SDA7.2.2. code[1, 2]. First, a set of partial atomic charges (net charges of the charged atom groups) are chosen to represent charge density of the solute. The OPLS parameter was used to assign atomic charges and radii to atoms. Then, the effective charges are generated via rescaling the partial atomic charges and fitted in such a way, that the electrostatic potential computed around the solute using those effective charges, surrounded by a shell with low dielectric constant and kept within a uniform solvent, replicates that of original reaction field, as obtained in case of the polarizable solvent. During the simulation the electrostatic interaction free energy between two solutes is estimated from the interaction between effective charges of a solute and the electrostatic potential grid surrounding the other. Next, the short range electrostatic

desolvation free energy between two solutes is modelled as interaction between effective charge of one solute and electrostatic desolvation potential of the other one. The non-polar desolvation interaction energy between two solutes is assumed to be proportional to the solvent accessible surface area of the solute that is obstructed by the other one. This interaction also depends on the non-polar burial surface potential of the second solute surrounding the first one. In biomolecules, prevention of overlapping between the molecules is assigned through excluded volume grids that are pre-computed around the molecule using a grid spacing of 1 Å. If, during a simulation step, one molecule penetrates the exclusion volume of the other molecule, then the move is regenerated with a different random number until it omits the overlapping.

The empirical scaling factor for weighing between long range electrostatic interaction energy and short range electrostatic desolvation term is set as 1.67 for our calculation. For non-polar desolvation, the potential depends on a scaling factor (γ) such that $\gamma = 1.0$ for all the points of the second solute, lying within a minimum distance (a =3.10 Å) from the surface of the first one, at this limit the surface of the first solute is completely occluded. Similarly, $\gamma = 0.0$ if the point is further than a maximum distance (b = 4.35 Å) from the surface of the first solute, where the presence of the second solute does not affect solvation of the first one. Finally, the factor is linearly interpolated if the distance lies in between these two limits. We use $\gamma = 0.5$ in our calculation as per observations obtained from trial and error method, to get better estimation of the potential. The surface of solute 1 that is excluded to solute 2 is computed by increasing radii of the atoms by 1.77 Å for proteins of solute 1, which have solvent accessible surface area (SASA) more than the threshold. A probe of radius 1.4 Å is used as a representative of solvent (water) molecule to calculate the SASA of the solute. Spacing of the exclusion grid is considered ~ 0.5 Å to take into account the shape of the solute. The entire

potential grid is multiplied by suitable factors to include the ionic contribution in potential grid.

Analysis protocol:

RMSD is being calculated using the following formula, $\mathrm{RMSD}(t) = \sqrt{\sum_{i=1}^{N} \frac{1}{N} |\boldsymbol{r}_i(\boldsymbol{t}) - \boldsymbol{r}_i(\boldsymbol{0})\,|^2}$, after aligning all the simulated structures of the molecule with respect to its initial conformation using script in VMD[3]. Here $\boldsymbol{r}_i(\boldsymbol{t})$ is the position of ith atom at time t, and $\boldsymbol{r}_i(\boldsymbol{0})$ is that for initial configuration. N is total number of atoms.

We use standard g_sasa command of GROMACS[4, 5] to generate SASA of system of interest ($SASA_{sys}$) over simulated data. A solvent representative sphere of radius r_{solv} is rolled over the envelop of the van der Waals surface of biotin, where this surface is composed of interlocking spheres of appropriate van der Waals radius consistent to the atom. Thus, SASA of an atom of radius r is defined as area of sphere with radius $R = r + r_{solv}$, given the fact the solvent sphere is in contact with the respective atom without penetrating any other atoms.

Binding kinetics calculations:

We also compute association rate constant between Streptavidin and relaxed conformation of our four different surfaces using SDA software[1, 2]. In this Brownian Dynamics based simulation, the protein is stochastically simulated by given small amount of displacement starting from the given configuration with respect to surface. The collision probability between protein and the surface at random spatial coordinate with different orientation can be obtained from the trajectory, which is finally being related to bio-molecular association rate constant. Escape probability as well as recombination probability after the collision is also taken into account. A sphere of radius b is chosen to divide configuration space of particle

pairs in such a way that for a separation r> b, hydrodynamic and columbic forces are assumed to be centro-symmetric and isotropic. We consider the same electrostatic potential, electrostatic desolvation and hydrodynamic desolvation grids for protein and surfaces that we used for docking. Other technical details for simulation are identical to those used for docking. We perform 5 different simulations, each for 2500 number of trajectories for proper sampling.

The association rate constant (Figure S5(a)) is plotted as a function of increasing distance between contact pairs of protein and surface that manifest binding. This indicates that the rate for both bPEG49 (k_{on}^{bPEG49}) and bPEG25-PEG24 ($k_{on}^{bPEG25-PEG24}$) are comparable ~ 1.02 $\times 10^{10}$ $M^{-1}sec^{-1}$, whereas for larger density system (k_{on}^{bPEG98}) and ($k_{on}^{bPEG49-PEG49}$) rates are smaller ~ 8.5×10^{9} $M^{-1}sec^{-1}$. Thus, from kinetic aspect, smaller density systems seem more prone to accumulate the protein. However, we also compute dissociation rate constant starting from the final docking orientations for all the surfaces. Here, the average resident time provide an indirect estimation of dissociation rate constant. Thus, larger the residence time, smaller the dissociation constant. Figure S5(b) shows that for bPEG98, residence time is larger up to a threshold distance between contact pairs ~ 10 Å, then for both bPEG49 and bPEG49-PEG49 residence times are comparable but lower than for bPEG98. The residence time is smaller for bPEG25-PEG24. We repeat the same analysis also for the final binding orientation between surfaces and protein as obtained after MD refinement. Here, also we find (Figure S5(c)) that larger the number of biotins, longer is the residence time and thus smaller is dissociation constant. Thus, we may conclude that while the association on a low density surface seem favourable, once the binding is formed, it takes longer time to dissociate the protein from surface layer for a system that has a larger number of biotins. This is similar to what we observe in MD simulation trajectories, i.e. for bPEG98 binding strength with Streptavidin is higher than bPEG49-PEG49, the strength is further reduced for bPEG49.

1. M. Martinez, N. J. Bruce, J. Romanowska, D. B. Kokh, M. Ozboyaci, X. Yu, M. A. Ozturk, S. Richter and R. C. Wade, *Journal of computational chemistry*, 2015, **36**, 1631-1645.
2. R. R. Gabdoulline and R. C. Wade, *Biophys J*, 1997, **72**, 1917-1929.
3. W. Humphrey, A. Dalke and K. Schulten, *Journal of molecular graphics*, 1996, **14**, 33-38, 27-38.
4. B. Hess, C. Kutzner, D. van der Spoel and E. Lindahl, *Journal of chemical theory and computation*, 2008, **4**, 435-447.
5. D. Van Der Spoel, E. Lindahl, B. Hess, G. Groenhof, A. E. Mark and H. J. Berendsen, *J. Comput. Chem.*, 2005, **26**, 1701-1718.

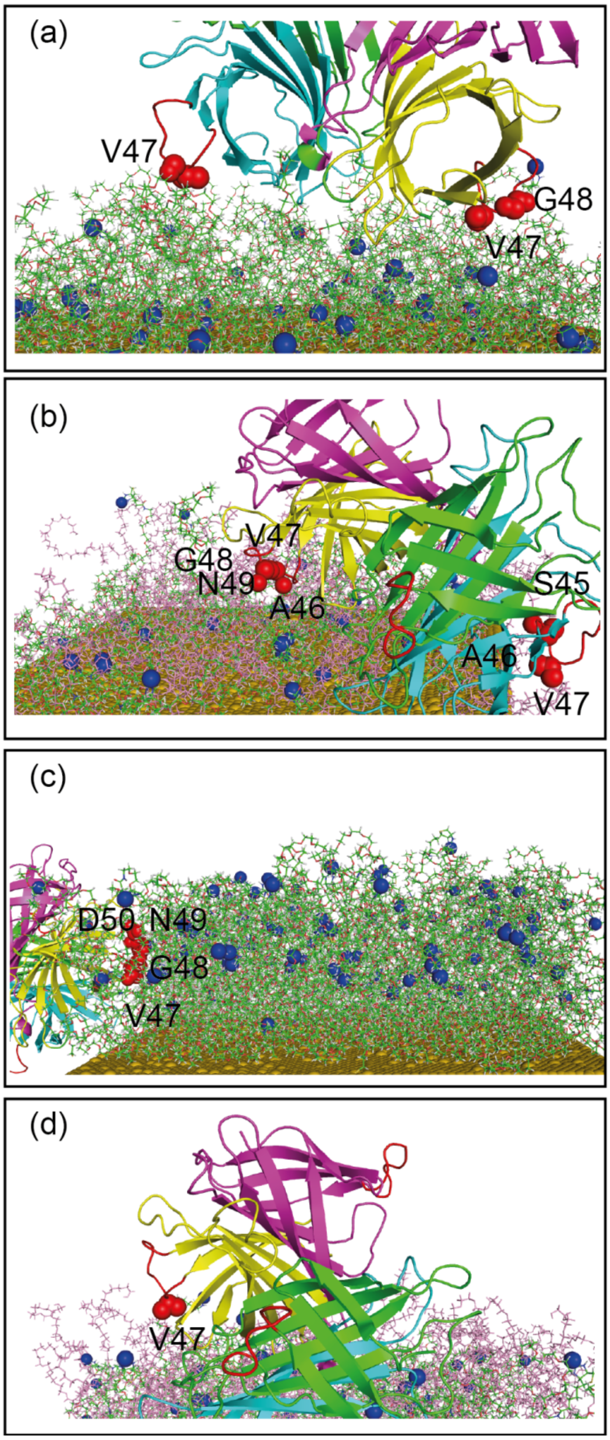

Figure S1. Zoom in final configuration of streptavidin in close proximity of four different surfaces, terminal biotin atoms are shown by spherical representation (blue), Au(111) in yellow, PEGs are in pink, bPEGs in green and protein is shown in cartoon representation with four different colours for tetramers, red spheres of proteins are the residues in proximity with bPEG/PEG in docked complex; (a) 49bPEG; (b) 25bPEG-24PEG; (c) 98bPEG; (d) 49bPEG-49PEG.

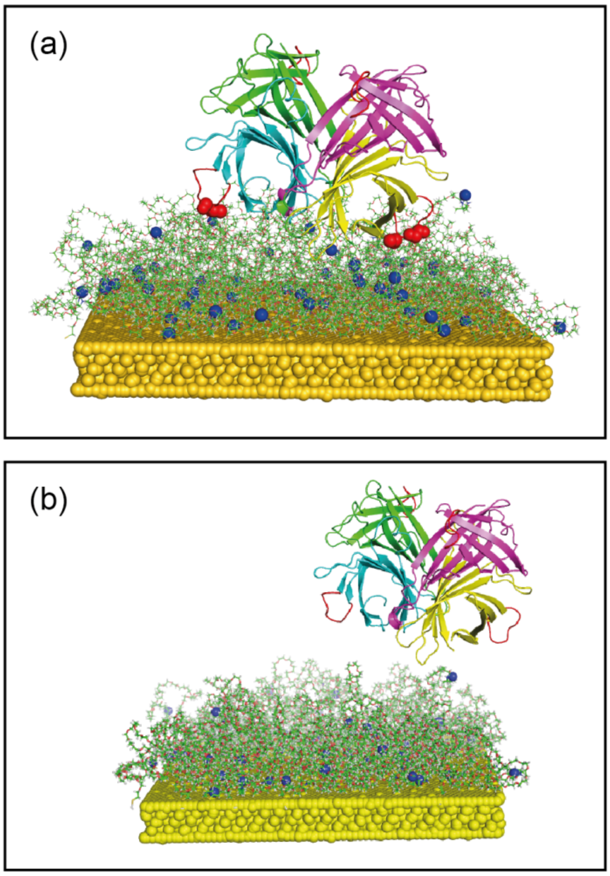

Figure S2 One representation for bPEG49, (a) docking pose of streptavidin over surface as obtained after SDA, (b) Initial configuration chosen for MD without altering docking orientation as obtained after SDA but giving protein translation ~50 Å from the surface.

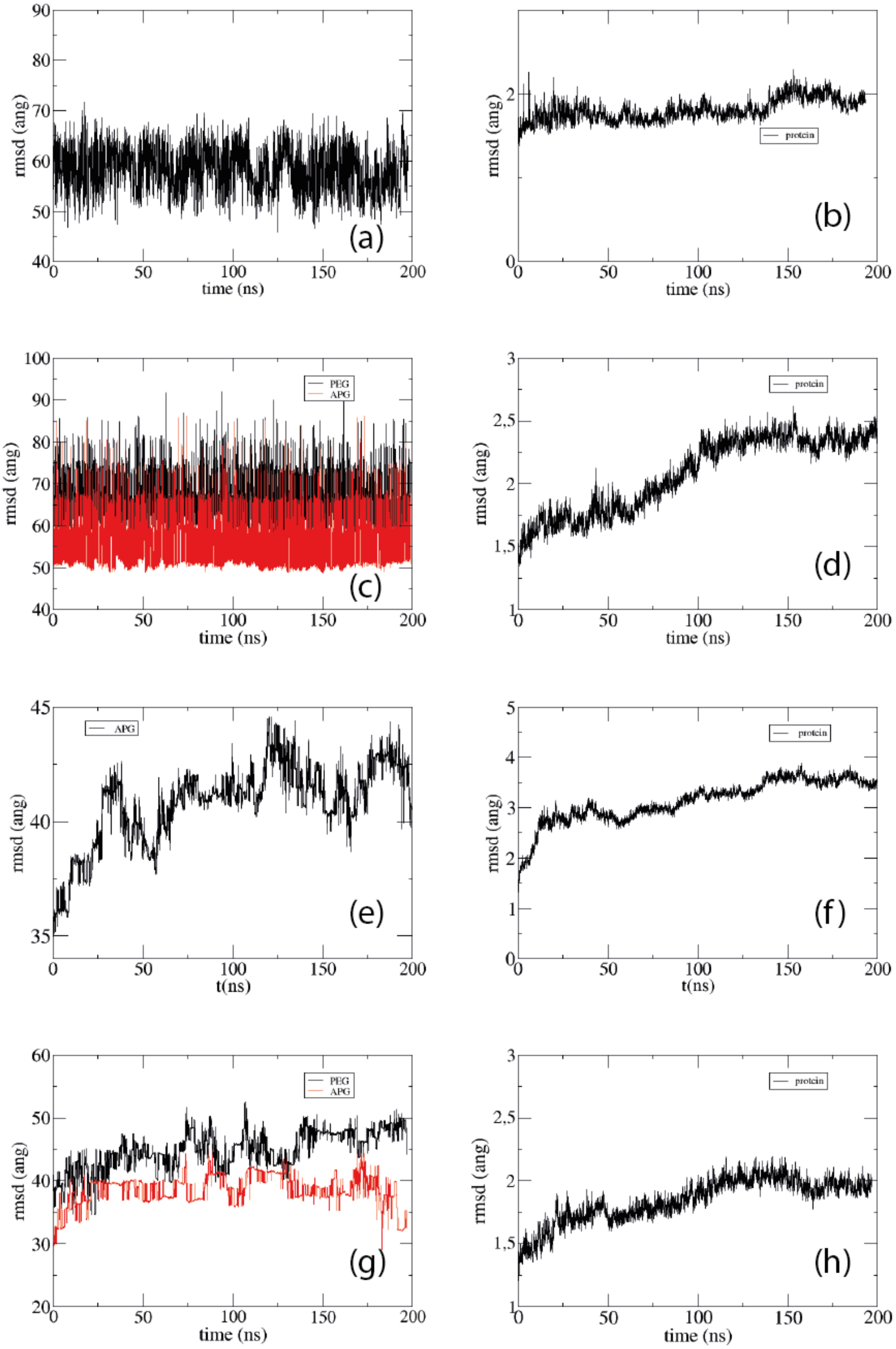
rmsd (ang)
time (ns)
(a)
protein
(b)
PEG
APG
(c)
(d)
t(ns)
(e)
(f)
(g)
(h)

Figure S3. RMSD plots generated over MD simulation trajectories. (a) PEGs (b) and that for protein in 49bPEG system, (c) PEG (black) and bPEG (red), followed by (d) for protein in 25bPEG-24PEG. (e) PEGs (f) and that for protein in 98bPEG system, (g) PEG (black) and bPEG (red), followed by (h) for protein in 49bPEG-49PEG.

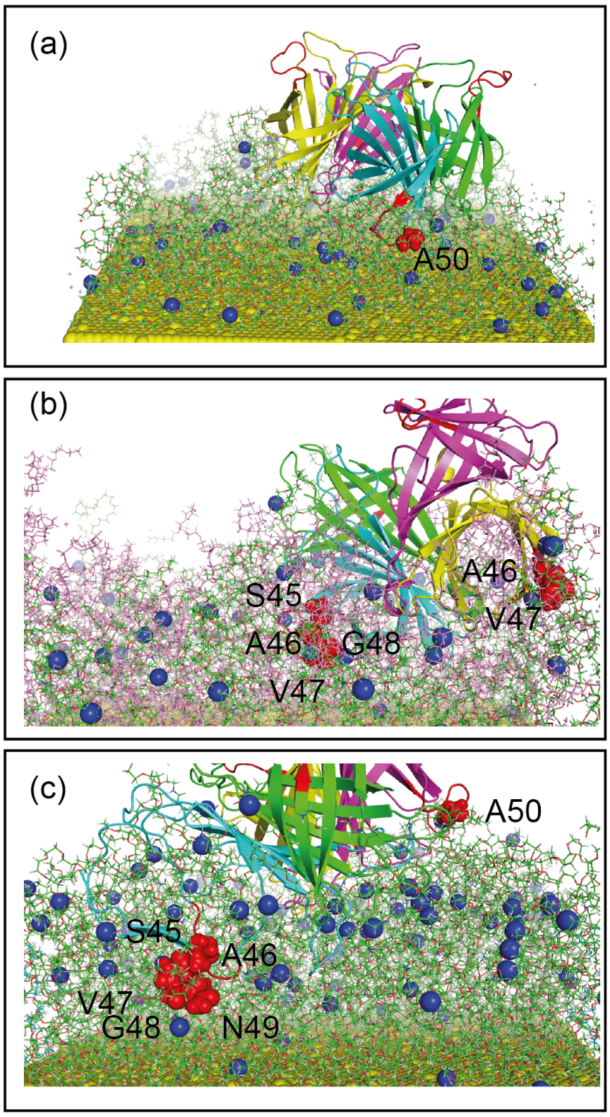

Figure S4. Streptavidin coming in proximity with different biotins via different biotin binding loops for different surfaces, terminal biotin atoms are shown by spherical representation (blue), Au(111) in yellow, PEGs are in pink, bPEGs in green and protein is shown in cartoon representation with four different colours for tetramers, red spheres of proteins are the residues in proximity with bPEG/PEG in docked complex; (a) 49bPEG; (b) bPEG49-peg49; (c) 98bPEG.

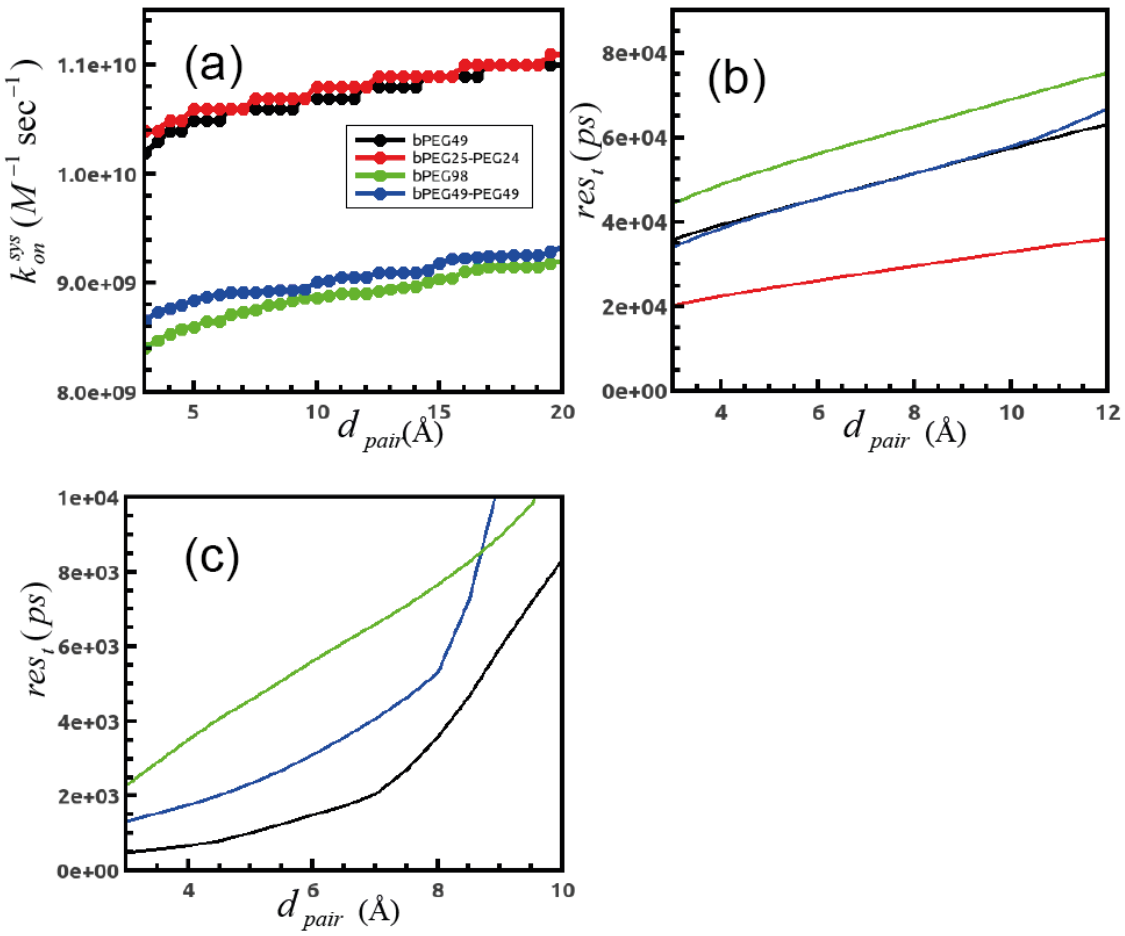

Figure S5. (a) Binding association rate constant for protein over different surface and (b) average residence time that can give indirect estimation of dissociation rate constant starting from docking orientation (c) estimation of dissociation rate for the final binding poses as obtained from MD.
